# Association between Hashimoto thyroiditis and clinical outcomes of papillary thyroid carcinoma: A meta-analysis

**DOI:** 10.1371/journal.pone.0269995

**Published:** 2022-06-16

**Authors:** Qizhi Tang, Weiyu Pan, Liangyue Peng

**Affiliations:** Department of Endocrinology, Guangdong Hospital of Integrated Traditional Chinese and Western Medicine, Foshan, P.R. China; Brigham and Women’s Hospital, Harvard Medical School, UNITED STATES

## Abstract

**Objective:**

To assess association between Hashimoto thyroiditis (HT) and clinical outcomes of papillary thyroid carcinoma (PTC).

**Methods:**

Databases including Pubmed, Embase, Cochrane Library, and Web of Science were searched. Weighed mean differences (WMDs) and odds ratios (ORs) were used to evaluate association between HT and clinical outcomes of PTC, and the effect size was represented by 95% confidence intervals (CIs). Heterogeneity test was performed for each indicator. If the heterogeneity statistic I^2^≥50%, random-effects model analysis was carried out, otherwise, fixed-effect model analysis was performed. Sensitivity analysis was performed for all outcomes, and publication bias was tested by Begg’s test.

**Results:**

Totally 47,237 patients in 65 articles were enrolled in this study, of which 12909 patients with HT and 34328 patients without HT. Our result indicated that PTC patients with HT tended to have lower risks of lymph node metastasis (OR: 0.787, 95%CI: 0.686–0.903, *P* = 0.001), distant metastasis (OR: 0.435, 95%CI: 0.279–0.676, *P*<0.001), extrathyroidal extension (OR: 0.745, 95%CI: 0.657–0.845, *P*<0.001), recurrence (OR: 0.627, 95%CI: 0.483–0.813, *P*<0.001), vascular invasion (OR: 0.718, 95%CI: 0.572–0.901, *P* = 0.004), and a better 20-year survival rate (OR: 1.396, 95%CI: 1.109–1.758, *P* = 0.005) while had higher risks of multifocality (OR: 1.245, 95%CI: 1.132–1.368, *P*<0.001), perineural infiltration (OR: 1.922, 95%CI: 1.195–3.093, *P* = 0.007), and bilaterality (OR: 1.394, 95%CI: 1.118–1.739, *P* = 0.003).

**Conclusions:**

PTC patients with HT may have favorable clinicopathologic characteristics, compared to PTCs without HT. More prospective studies are needed to further elucidate this relationship.

## Background

Hashimoto thyroiditis (HT) is a chronic inflammation of the thyroid gland initially described over a century ago, which is now considered the most common autoimmune disease [1, 2]. An incidence is estimated to range from 0.3 to 1.5 cases per 1,000 people, with a prevalence of 5–10% in the overall population [3]. HT is characterized by hypothyroidism, the presence of serum antithyroglobulin and antiperoxidase antibodies, and widespread lymphocytic infiltration with depletion of follicular cells [4, 5]. Thyroid cancer (TC) is the most common malignancy of the endocrine system, with papillary thyroid carcinoma (PTC) being the most prevalent form that accounts for 80% of all diagnosed TCs [6]. The incidence of PTC and HT is rapidly increasing in many countries [7, 8]. The disease of PTC coexisted with HT presents an increasing trend year by year [9]. The coexistence of these two diseases has also been reported to range from 10% to 58% [10, 11], which has aroused great concern.

The relationship between HT and PTC was investigated in several studies. Coexistent HT has been reported to be significantly associated with the less aggressive clinicopathologic characteristics of PTC [10, 12]. Whereas several scholars observed HT is associated with a significantly increased risk of PTC [13]. Other studies have shown no connection between the presence of HT and PCT [14, 15]. Moreover, the association with prognosis between HT and PC remains unclear. It is uncertain whether coexisting with HT in PTC represents a good prognosis or is simply the concurrence of both diseases. It is therefore reasonable to further evaluate the association between HT and PTC.

Herein, we conducted a meta-analysis with a multitude of outcome assessments included to explore the association between HT and PTC prognosis.

## Methods

### Search strategy

Published literature search was performed on Pubmed, Embase, Cochrane Library, and Web of Science databases from inception to December 11, 2020. The search words were as follows: “Thyroid Cancer, Papillary” OR “Cancer, Papillary Thyroid” OR “Papillary Thyroid Cancer” AND “Hashimoto Disease” OR “Hashimoto Struma” OR “Hashimoto Thyroiditis” OR “Hashimoto Thyroiditides” OR “Autoimmune thyroid disease”. The detailed search terms from PubMed are listed in S1 File.

### Inclusion and exclusion criteria

Inclusion criteria were: (1) studies with patients with PTC; (2) studies including patients with HT in the case group, and those without HT in the control group; (3) studies with the latest research results for the same studies by the same authors; (4) studies published in English; (5) cohort studies, case-control studies, and cross-sectional studies.

Exclusion criteria: (1) animal experiments; (2) studies in which data were incomplete; (3) reviews, meta-analyses, case reports, conference reports, editorial materials, and letters.

### Quality assessment and data extraction

The Chinese version of the Newcastle-Ottawa Scale (NOS) was used to evaluate the quality of the literature in cohort studies and case-control studies. The total score of the scale was 10, with < 5 as low quality and ≥5 as high quality. Regarding quality evaluation of cross-sectional studies, the Business Integration (JBI) scale was adopted, with 1–14 as low quality and 15–20 as high quality.

For each study, the following information was extracted, including author, year, country, study design, group, the number of patients, gender, age, subtype, tumor size, extent of surgery, tumor node metastasis stage, follow up, quality, outcomes.

### Outcomes

The association between HT and clinical outcomes of PTC was assessed by lymph node metastasis (including lymph node metastasis, central lymph node metastasis, lateral lymph node metastasis), distant metastasis, extrathyroidal extension, recurrence, multifocality, invasion (includes vascular invasion, capsular invasion, perineural infiltration), bilaterality, number of deaths, AMES stage and MACIS score.

### Statistical analysis

Software Stata (version 15.1, Stata Corporation, College Station, TX, USA) was used for statistical analysis. Weighed mean differences (WMDs) were statistics for measurement data, odds ratios (ORs) were used as effect indicators for continuous variables and frequency of events, and effect sizes were represented by 95% confidence intervals (CIs). A heterogeneity test was performed for each indicator. If THE heterogeneity statistic I^2^≥50%, random-effects model analysis was carried out, otherwise, fixed-effects model analysis was performed. Each meta-analysis may create a false-positive or negative conclusion. Given this, TSA was conducted to reduce these statistical errors [16]. TSA is a methodology that combines an information size calculation (accumulated sample sizes of all included trials) to reduce type I error and type II error for a meta-analysis with the threshold of statistical significance (http://www.ctu.dk/tsa). TSA was used to quantify the statistical reliability of data in the cumulative meta-analyses by adjusting significance levels for sparse data and repetitive testing on accumulating data. Sensitivity analysis was performed for all outcomes, and publication bias was tested by Begg’s test. Given the age imbalance between the case group and control group, an age-based sensitivity analysis was also applied (S2 File). *P*<0.05 was considered statistically significant.

## Results

**Fig 1:**
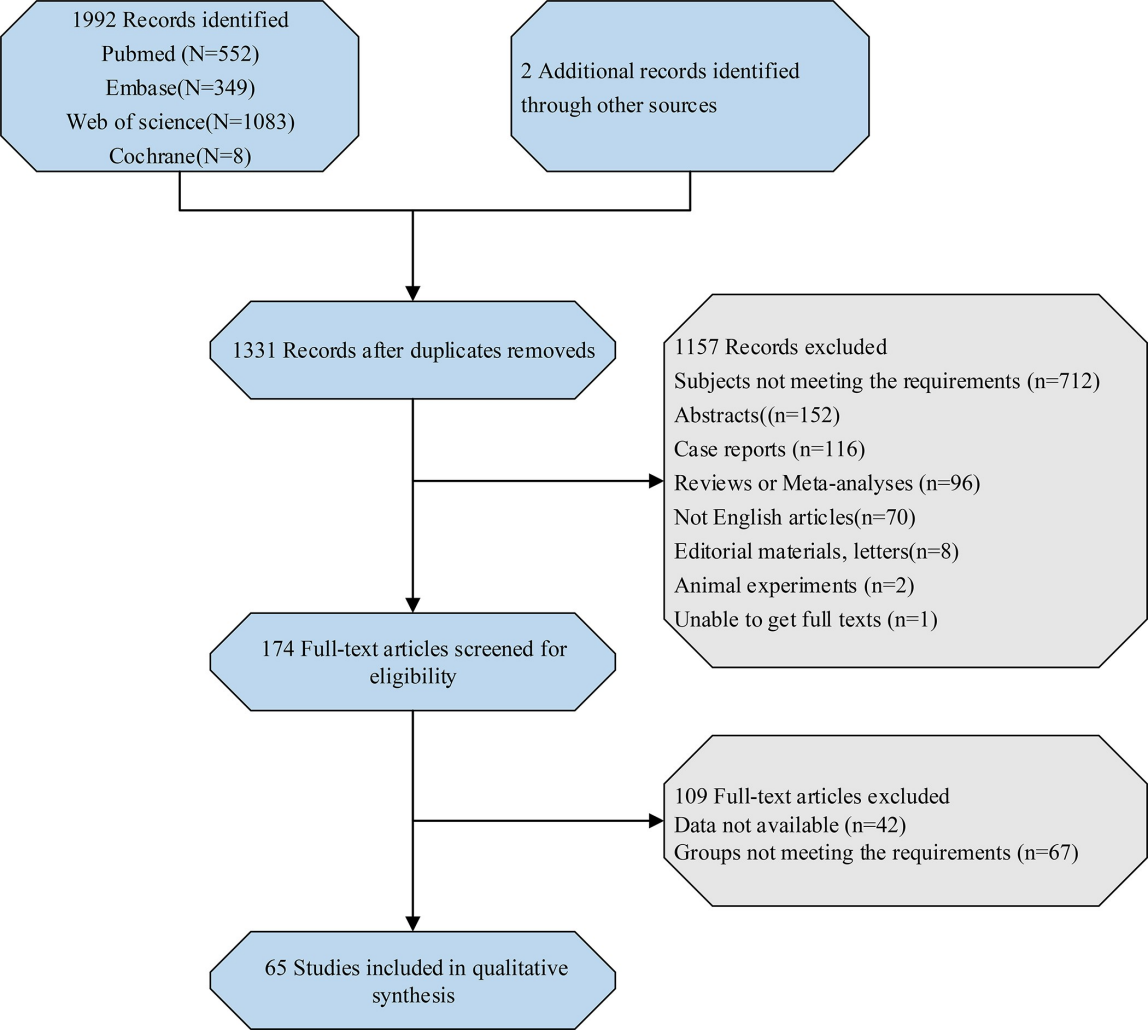
Flow chart of the study selection process.

Initially, 1992 studies were searched according to the search strategy, and after duplicated removed, 1331 records were identified. With 174 full-text articles eligible for screening, 65 articles [5, 8, 9, 12, 13, 17–76] were finally included in this meta-analysis, including 32 case-control studies, 27 cohort studies, and 6 cross-sectional studies. The flow chart depicting the study selection process is shown in Fig 1. Totally 47,237 patients were enrolled in this study, of which 12909 patients with HT and 34328 patients without HT. The characteristics of included studies are presented in Table 1.

**Table 1:** Basic characteristics of included studies.

Author	Year	Country	Study design	Group	Diagnosis of HT	No	Sex(female/male)	Age	Subtype of PTC	Tumor size (cm)	Extent of surgery	TNM stage	Follow up (months)	QA	Outcomes
Ahn	2011	Korea	retrospective cohort	PTC only	-	211	170/41	48.32±14.4	conventional 203, follicular variant 7, tall cell variant 1	1.8±1.5	TT 178, thyroid lobectomy with isthmusectomy 33	I 127, II/III/IV 84	62.8±27.0	9	①③④⑤⑧⑨⑩
				PTC+HT	diffuse lymphoplasmacytic infiltration, germinal centres, and enlar ged epithelial cells with large nuclei and eosinophilic cytoplasm (Askanazy or Hurthle cells)	58	55/3	42.8±12.7	conventional 55, follicular variant 2, tall cell variant 1	1.6±1.0	TT 47, thyroid lobectomy with isthmusectomy 11	I 35, II/III/IV 23	59.0±25.4		
Babli	2018	Canada	retrospective cohort	PTC only	-	309	258/58	49.3±13.87	-	2.06±1.62	TT 475	I 192, II 47, III 51, IV 19	25.5±19.5	7	①②③④⑤⑥⑦⑨
				PTC+HT	diffuse lymphoplasmacytic and plasma cell infiltration, lymphoid follicle formation with ger minal centers, varying degree of fibrosis, parenchymal atrophy, and the presence of large follicular cells with abundant oxyphilic cell changes	166	146/20	48±13.78		1.96±1.53		I 120, II 16, III 24, IV 6	24.6±16.3		
Bircan	2014	Turkey	retrospective case-control	PTMC only	-	105	81/24	51.06±13.24	papillary 70, FVPC 27, follicular 8	< 0.5 58	-	-	-	5	①⑥⑦
				PTMC+HT	diffuse lymphoplasmacytic infiltration, germinal centres, and enlarged epithelial cells with large nuclei and eosinophilic cytoplasm (Askanazy or Hurthle cells)	67	62/5		papillary 38, FVPC 27, follicular 2	< 0.5 35					
Cai	2015	China	retrospective case-control	PTC only	-	823	827/225	46.2±11.4	-	1.1±0.8	TT or lobectomy with prophylactic CLND and/or therapeutic LLND	I/II 753, III/IV 299	-	5	①③⑤
				PTC+HT	diffused lymphoplasmacytic infiltration with germinal centers, parenchymal atrophy with oncocytic changes, and variable amounts of stromal fibrosis throughout the thyroid gland	229				1.1±0.8					
Carvalho	2017	Brazil	prospective cohort	PTC only	-	442	347/95	median 46 (14–76)	-	≤2 132, 2–4 228, >4 82	TT 633	T1bN0 35, T1N1 62, T2N0 87, T2N1 53, T3N0 119, T3N1 80, T4N0 1, T4N11	66 (24–120)	8	①③④⑥⑦
				PTC+HT	histological criteria included diffuse lymphoplasmacytic infiltration	191	160/31	median 48 (13–80)		≤2 62, 2–4 96, >4 33		T1bN0 18, T1N1 24, T2N0 38, T2N1 24, T3N0 52, T3N1 35			
Consorti	2010	Italy	retrospective case-control	PTC only	-	76	57/19	56.27±12.79	-	1.265±1.203	TT 101	I 57, II 3, III/IV 16	-	6	①⑥
				PTC+HT	dense focal or diffuse lymphocytic and plasma cell infiltration of the thyroid, with formation of lymphoid follicles including germinal centres, follicular hyperplasia and damage to the follicular basement membrane	25	20/5	54.48±13.37		1.571±1.271		I 15, II 1, III/IV 9			
Cordioli	2013	Brazil	retrospective case-control	PTC only	-	59	49/10	44.7±14.7	-	2.51±2.09	-	T1/T2 26, T3/T4 33	-	6	①⑤⑦
				PTC+HT	diffuse lymphocytic and plasma cell infiltrate with the presence of lymphoid follicles with reactive germinal centers, as well as occasional Hürthle cells	35	31/4	45.8±13.2		1.56±1.30		T1/T2 23, T3/T4 12			
Cortes	2018	Brazil	prospective cohort	PTC only	-	68	61/7	median 48 (18–74)	-	-	TT 113	I 40, II 6, III 14, IV 8	96 (62–140)	7	①②
				PTC+HT	diffuse lymphoplasmacytic infiltration	45	42/3	median 46 (13–72)				I 24, II 4, III 9, IV 8	96(60–140)		
Dobrinja	2016	Italy	retrospective cohort	PTC only	-	90	56/34	54 (12–84)	-	median 1.34 (0.05–6.5)	TT 85, loboistmectomy 5	I 55, II 3, III 21, IV 11	39 (18–343)	8	①④⑤⑧
				PTC+HT	laboratory tests and postsurgical histological examination, histopathological criteria included epithelial cell destruction and mononuclear lymphocytic infiltrate accompanied by lymphoid germinal center formation and variable degree offibrosis	70	61/9	52 (19–86)		median 1.54 (0.06–10.0)	TT 68, loboistmectomy 2	I 50, II 4, III 14, IV 2	47 (18–156)		
Dvorkin	2013	Israel	retrospective cohort	PTC only	-	98	90/8	50.5±15	-	1.95±1.3	TT 196	I 55, II 11, III 23, IV 9	≥12	8	③⑤
				PTC+HT	patient’s history of hypothyroidism with positive antithyroid antibodies or when there was a diffuse lymphocytic infiltration bilaterally on the pathology report	98	91/7	50.5±15		1.78±1.2		I 57, II 9, III 22, IV 8			
Fiore	2011	Italy	cross-sectional	PTC only	-	554	405/149	39.6±13.1	classic 312, tall cell 73, follicular 80, mixed form 79, other 10	-	-	T1 211, T2 72, T3 271	-	13	①
				PTC+HT	a) high titer of TAb (>100 U/ml of both TgAb and TPOAb) or hypothyroid, or b) positive TAb not fulfilling the criteria reported in point a), but presented a clear hypoechoic ‘thyroiditis’ pattern at thyroid ultrasound	112	91/21	-	classic 59, tall cell 19, follicular 16, mixed form 16, other 1			T1 46, T2 13, T3 53			
Giagourta	2014	Greece	retrospective cross-sectional	PTC only	-	939	817/122	54±14	papillary 610, papillary/follicular 329	2.12±1.62	TT 1380	I 410, II 364, III/IV 165	-	15	①②⑤⑦
				PTC+HT	dense or diffuse lymphocytic and plasma cell infiltration, oxyphilic cells, and formation of lymphoid follicles in the tissue of both lobes	441	379/62	42±10	papillary 282, papillary/follicular 159	1.83±1.53		I 206, II 190, III/IV 45			
Girardi	2015	Brazil	retrospective cross-sectional	PTC only	-	269	203/66	47.15±14.14	-	1.91±1.70	TT 269, lymphadenectomy 102	I 181, II 10, III 63, IV 15	-	14	①②③⑤⑥
				PTC+HT	an association of lymphoplasmacytic infiltration with germinative center formation, oxyphilic cell metaplasia (Hürtle), atrophy, and fibrosis of thyroid follicles	148	136/12	45.97±14.10		1.40±1.15	TT 148, lymphadenectomy 79	I 119, II 5, III 22, IV 2			
Han	2019	China	case-control	PTC only	-	89	63/26	42.3±12.3	-	1.2±0.7	-	I/II 88, III/IV 1	-	4	①③⑤
				PTC+HT	pathological diagnosis	49	47/2	39.9±12.5		1.3±0.9		I/II 47, III/IV 2			
Huang	2011	China	retrospective cohort	PTC only	-	1703	1366/337	40.8±14.2	-	2.3±0.04	TT 411, LND/radical neck dissection 1292	I 1278, II 140, III 76, IV 205	116.4±2.4	7	④⑧
				PTC+HT	histological diagnosis	85	84/1	39.5±12.6		2.1±0.1	TT 14, LND/radical neck dissection 71	I 70, II 4, III 7, IV 4	104.4±7.2		
Ieni	2017	Italy	retrospective case-control	PTC only	-	337	253/84	47.21±13.76	classic variant 180, follicular variant 118, sclerosing 23, tall cell 6, Warthin-like 3, hobnail/micropapillary 6, cribriform 1	1.2±0.971	-	T1a 168, T1b 67, T2 22, T3 79, T4 1	-	5	①
				PTC+HT	lymphocytic infiltration with germinal center formation and Hürthle cell metaplasia	168	146/22	44.42±13.72	classic variant 76, follicular variant 65, sclerosing 16, tall cell 3, Warthin-like 6, hobnail/micropapillary 2	0.939±0.61		T1a 110, T1b 38, T2 12, T3 8			
Jara	2013	USA	retrospective case-control	PTC only	-	269	192/77	median 47(11–86)	conventional 205, follicular variant 48, tall cell variant 16	2.08(1.0–2.5) *	TT 257, hemithyroidectomy 12	I 169, II 13, III 55, IV 32	-	6	①③⑤⑦
				PTC+HT	pathological diagnosis	226	199/27	median 43(17–80)	conventional 159, follicular variant 45, tall cell variant 18, trabecular variant 3, warthin-like features 1	1.56(0.8–2.0) *	TT 219, hemithyroidectomy 7	I 180, II 7, III 23, IV 16			
Jeong	2012	Korea	retrospective cohort	PTC only	-	402	332/70	48.56±11.02	-	1.12±0.77	TT 402	I 227, II 2, III 163, IV 10	58.12±8.12	8	①③④⑤⑨⑩
				PTC+HT	diffuse lymphoplasmacytic infiltrate, oxyphilic cells, formation of lymphoid follicles with germinal centers and atrophic changes in the area of normal thyroid tissue	195	188/7	47.24±9.84		0.99±0.61	TT 195	I 107, III 84, IV 3	58.55±7.19		
Kashima	1998	Japan	case-control	PTC only	-	1252	1123/129	48.6	-	2.82	-	-	82.8±56.4	6	①③⑤⑦
				PTC+HT	obvious lymphoid follicles with germinal centers and coexisting atrophie follicular epithelium	281	279/2	42.6		2.42			127.2±72		
Kebebew	2001	USA	retrospective cohort	PTC only	-	95	61/34	54	-	≤1 37, 1–4 44, >4 7, unknown 7	TT/near TT 64, subtotal thyroidectomy 5, lobectomy 26	I 61, II 12, III 13, IV 5	52.8	8	⑥⑧⑩
				PTC+HT	diffuse lymphoplasmacytic infiltrate, oxyphilic cells, formation of lymphoid follicles with germinal centers and atrophic changes in the area of normal thyroid tissue	41	34/7	45.5		≤1 19, 1–4 19, >4 1, unknown 2	TT/near TT 26, subtotal thyroidectomy 4, lobectomy 11	I 27, II 6, III 6			
Kim	2009	Korea	case-control	PTC only	-	64	54/10	44.1±13.4	-	1.48±1.13	-	I/II 38, III/IV 26	-	6	①③
				PTC+HT	diffuse lymphoplasmacytic infiltration with germinal centers, parenchymal atrophy with oncocytic change, and variable amounts of stromal fibrosis throughout the thyroid gland	37	36/1	49.4±12.7		1.22±0.88		I/II 17, III/IV 20			
Kim	2010	Korea	case-control	PTMC only	-	218	174/44	<45 81	-	0.8–1 100	TT 128	-	-	6	③⑤⑥
				PTMC+HT	heavy infiltration of lymphocytes with varying degrees (including germinal centers) in thyroid tissue, the presence of Hurthle cells and varying degree of acini atrophy	105	100/5	<45 37		0.8–1 46	TT 57				
Kim	2011	Korea	case-control	PTC only	-	254	219/35	48.1±11.5	-	1.13±0.86	TT 397, bilateral CLND 395, lateral or modified LND 95	I/II 125, III/IV 129	-	6	①②③⑤⑦
				PTC+HT	any 1 of the following criteria: (1) positive for anti-TPO antibody, (2) positive for antithyroglobulin antibody, (3) pathologic confirmation of Hashimoto’s thyroiditis	146	138/8	45.9±11.6		1.10±0.64		I/II 88, III/IV 58			
Kim	2011	Korea	case-control	PTC only	-	721	527/194	48.0±12.1	-	1.24±0.96	-	III/IV 312	-	6	①③⑤
				PTC+HT	diffuse lymphoplasmacytic infiltrations with germinal centers, parenchymal atrophy with oncocytic changes, and variable amounts of stromal fibrosis throughout the thyroid gland	307	294/13	47.5±10.3		1.08±0.72		III/IV 118			
Kim	2013	Korea	retrospective cohort	PTC only	-	931	778/153	46.80±11.01	-	1.01±0.75	lobectomy 106, TT+MRND 824	-	-	7	③④⑤⑦
				PTC+HT	diffuse lymphoplasmacytic infiltrate, oxyphilic cells, formation of lymphoid follicles with germinal centers and atrophic changes in the area of normal thyroid tissue	316	304/12	46.41±10.48		0.93±0.63	lobectomy 19, TT+MRND 297				
Kim	2014	Korea	retrospective cohort	PTC only	-	125	114/11	48.6(25–75)	-	0.93(0.2–4)	TT 144	-	68.9±8.1	8	①③④⑤
				PTC+HT	diffuse lymphocytic thyroiditis with follicular atrophy, diffuse destruction of thyroid follicles, fibrosis, and follicular cell regeneration	19	19/0	43.0(28–65)		0.86(0.3–1.5)					
Kim	2016	Korea	case-control	PTC only	-	1576	1289/287	47.2±12.0	-	0.9±0.6	TT 1466, <TT 110	I/II 957, III/IV 619	-	5	①③⑤
				PTC+HT	diffuse lymphoplasmacytic infiltration with germinal centers, parenchymal atrophy with oncocytic changes, and variable amounts of stromal fibrosis throughout the thyroid gland	204	198/6	44.8±11.9		0.8±0.5	TT 190, <TT 14	I/II 149, III/IV 55			
Kim	2016	Korea	case-control	PTC only	-	2326	1687/639	47.6±11.9	-	1.2±0.8	TT+bilateral CND 3332	-	-	6	①③⑥
				PTC+HT	diffuse parenchymal infiltration by lymphocytes (particularly plasma B-cells), a germinal center formation, follicular destruction, Hurthle cell change and variable amounts of stromal fibrosis throughout the thyroid gland	1006	912/94	46.0±11.4		1.1±0.7					
Kim	2018	Korea	retrospective case-control	PTC only	-	124	107/17	50.06±11.51	-	0.92±0.73	TT 172	I/II 71, III 40, IV 13	-	6	①③⑤⑥
				PTC+HT	diffuse lymphocytic and plasma cell infiltrate, oxyphilic cells and the formation of lymphoid follicles or reactive germinal centers in the area of normal thyroid tissue	48	45/3	46.44±10.62		0.88±0.69		I/II 36, III 11, IV 1			
Konturek	2014	Poland	retrospective cohort	PTC only	-	643	574/69	<45 278, ≥45 365	-	0.94±0.69	TT+CLND, subtotal bilateral lobectomies	T1a 391, T1b 57, T2 78, T3 108	-	7	①⑤
				PTC+HT	(1) high anti-thyroid peroxidase antibodies titers(anti-TPO), (2) lesions visualized by ultrasonography showing a hypoechoic or hyperechoic nodular pattern at least 5 mm in diameter, identification of a perinodular hypoechogenic or hyperechogenic halo and presence of an anechoic lesion with a reinforced posterior wall, (3) histology: presence of a diffuse lymphocytic infiltrate in the thyroid parenchyma and stroma with reaction foci and lymphatic follicles, presence of small follicles with a decreased colloid volume, foci of fibrosis and oxyphilic cytoplasm-containing cells	130	110/20	<45 52, ≥45 78		0.87±0.59		T1a 80, T1b 22, T2 12, T3 16			
Kurukahvecioglu	2007	Turkey	retrospective case-control	PTC only	-	162	123/39	46.6±13.5	follicular variant 37	<1 18, ≥1 114	TT 199	-	-	6	⑥
				PTC+HT	diffuse mononuclear cell infiltration with fibrosis, occasional well-developed germinal centers, and enlarged follicular cells with abundant eosinophilic, granular cytoplasms	37	36/1		follicular variant 4	<1 19, ≥1 48					
Kwak	2015	Korea	retrospective cohort	PTC only	-	1493	1187/306	46.12±12.13	classical 1369, follicular variant 84, cystic 14, oncocytic 4, others 22	0.935±0.7	thyroid lobectomy or TT with cervical LND 1945	I 648, II 9, III 736, IV 100	27(9–55)	8	③④⑤⑦⑧
				PTC+HT	pathological diagnosis included chronic lymphocytic infiltration	452	412/40	45.25±11.63	classical 412, follicular variant 31, cystic 4, oncocytic 1, cribriform 1, others 3	0.003±0.762		I 229, II 2, III 205, IV 16			
Kwon	2014	Korea	cohort	PTC only	-	86	72/14	48.8±12.2	conventional 79, variants 7	<2 70, 2–4 15, >4 1	-	-	64.8±8.6	7	①③⑤⑦
				PTC+HT	diffuse lymphoplasmacytic infiltration with germinal centers, parenchymal atrophy with oncocytic change, and variable amounts of stromal fibrosis throughout the thyroid gland	84	80/4	47.1±11.6	conventional 71, variants 13	<2 76, 2–4 6, >4 2			64.3±11.1		
Kwon	2016	Korea	retrospective cohort	PTC only	-	473	350/123	48.4±10.5	-	1.23±0.93	TT+CLND 433	-	69.1±23.5	5	①③⑤⑥⑦
				PTC+HT	histological diagnosis	215	200/15	46.9±10.4		1.11±0.96	TT+CLND 198				
Lee	2018	Korea	case-control	PTC only	-	1296	967/329	47.3±12.0	-	0.83±0.53	-	-	-	5	①③⑤
				PTC+HT	pathology reports or chronic lymphocytic thyroiditis	563	528/35	46.4±11.3		0.83±0.58					
Lee	2020	Korea	retrospective cohort	PTC only	-	1754	1286/468	46.4±0.28	-	1.06±0.022	TT 854, <TT 900	T1 1426, T2 86, T3 184, T4 58	24(1–90)	5	①③④⑤⑥⑦
				PTC+HT	lymphoplasmacytic infiltration with germinal center and the presence of large follicular cells with abundant granular eosinophilic cytoplasm on histologic examination	1174	1082/92	45.5±0.32		0. 96±0.021	TT 822, <TT 352	T1 918, T2 46, T3 189, T4 21			
Liang	2017	China	retrospective cohort	PTC only	-	1035	789/246	45.34±12.63	-	1.94±1.14	thyroid lobectomy with isthmusectomy 520, TT 872, CLND without LLND 785, comprehensive neck dissections 495	I 644, II 51, III 175, IV 165	38.4 (3.1–125.3)	8	①②③④⑤⑧⑨⑩
				PTC+HT	diffuse lymphoplasmacytic infiltration, germinal centres and enlarged epithelial cells with large nuclei and eosinophilic cytoplasm	357	323/34	44.14±11.95		1.58±0.97		I 252, II 7, III 67, IV 31			
Lim	2013	Korea	retrospective case-control	PTC only	-	1983	1476/507	median 45	-	0.83	bilateral TT 2316, unilateral TT 751	III/IVA 698	-	4	①③⑤
				PTC+HT	pathology reports	964	873/91	median 45		0.79		III/IVA 311			
Liu	2014	China	retrospective case-control	PTC only	-	1141	840/301	45.25±13.63	-	1.508±0.0358	-	-	-	6	①⑥
				PTC+HT	diffuse lymphocytic infiltration with the formation of lymphoid follicles and reactive germinal centers	581	535/46	41.40±13.26		1.392±0.0421					
Liu	2016	China	retrospective case-control	PTMC only	-	119	77/42	46.35±11.23	-	0.87±0.22	-	-	-	5	①⑤⑦
				PTMC+HT		49	38/11	42.18±9.84		0.65±0.12					
Lu	2020	China	case-control	PTC only	-	89	63/26	42.6±12.4	-	1.1±0.7	-	I/II 88, III/IV 1	-	4	①③⑤
				PTC+HT	pathology reports	51	47/4	39.5±13.1		1.4±1.0		I/II 49, III/IV 2			
Lun	2013	China	case-control	PTC only	-	549	419/130	44.8±13.3	-	2.24±1.38	-	III/IV 101	-	6	①
				PTC+HT	diffuse lymphocytic infiltration, germinal centers, enlarged epithelial cells with large nuclei and eosinophilic cytoplasm (Askanazy or Hurthle cells), and variable amounts of stromal fibrosis throughout the thyroid gland	127	118/9	41.3±12.5		1.84±0.93		III/IV 8			
Ma	2018	China	case-control	PTC only	-	365	306/59	<45 180, ≥45 185	-	<1 150, ≥1 215	-	-	-	4	①
				PTC+HT		85	79/6	<45 40, ≥45 45		<1 41, ≥1 44					
Marotta	2013	Italy	case-control	PTC only	-	92	66/26	56.1	-	1.12ml	-	I 54, II 19, III 19	-	6	①③⑤
				PTC+HT	lymphoplasmacytic infiltrations with germinal centers, and serum antithyroperoxidase antibodies measured by an immunoenzymatic assay	54	50/4	50.2		0.84ml		I 36, II 4, III 14			
Marotta	2017	Italy	multicentre retrospective cohort	PTC only	-	173	133/40	median 37 (15–71)	classic 96, follicular 36, hürthle cells 9, warthin-like 6, tall cell 12, solid 9, diffuse sclerosing 5	median 1.3 (0.7–4)	-	-	75±59	8	⑤
				PTC+HT	diffuse/focal lymphoplasmacytic infiltrate, oxyphilic cells, lymphoid follicles with germinal centres and atrophic changes involving normal thyroid tissue	128	120/8	median 39.5 (17–64)	classic 61, follicular 42, hürthle cells 5, warthin-like 18, tall cell 1, solid 1	median 1 (0.6–4)					
Mohamed	2020	Egypt	cross-sectional	PTC only	-	64	44/20	≤ 45 24, > 45 40	follicular variant 24, classic variant 40	≤ 2 38, > 2 26	TT or near TT 80	I/II 44, III/IV 20	120 (84–120)	15	①②③④⑤⑥⑧
				PTC+HT	lymphoplasmacytic infiltration with the formation of germinal center, oxyphilic cell metaplasia (Hürthle cells), atrophy, and fibrosis of thyroid follicles	16	14/2	≤ 45 6, > 45 10	follicular variant 4, classic variant 12	≤ 2 12, > 2 4		I/II 10, III/IV 6			
Molnar	2019	Hungary	case-control	PTC only	-	190	164/26	48.03±16.74	classic 143, follicular variant 34, other 13	-	-	1.33±0.79	-	7	①⑤
				PTC+HT	chronic lymphocytic infiltration, secondary lymphatic follicules and follicular atrophy, occasionally extended by the additional presence of Hürthle cell metaplasia	40	36/4	44.03±16.18	classic 28, follicular variant 9, other 3			1.20±0.61			
Nam	2016	Korea	retrospective cohort	PTC only	-	15	10/5	47.13±13.84	-	median 1.4 (0.7–4.5)	TT + unilateral/bilateral CLND 37	I 5, II 1, III 4, IV 5	51.81±16.35	8	①②③⑤
				PTC+HT	diffuse lymphoplasmacytic infiltrate, oxyphilic cells, formation of lymphoid follicles with germinal centres and atrophic changes in the area of normal thyroid tissue	22	21/1	44.18±13.64		median 1.1 (0.3–3.5)		I 14, III 6, IV2	47.65±14.45		
Park	2015	Korea	retrospective case-control	PTC only	-	484	401/83	46.06±10.56	-	1.055±0.715	TT 294, lobectomy 153, subtotal thyroidectomy 37	T1a 219, T1b 59, T2 11, T3 192, T4 2	-	6	①⑤⑥
				PTC+HT	a progressive loss of thyroid follicular cells with replacement by lymphocytes and formation of germinal centers associated with fibrosis	49	48/1	43.80±9.92		0.875±0.398	TT 38, lobectomy 10, subtotal thyroidectomy 1	T1a 30, T1b 9, T3 10			
Paulson	2012	USA	historical cohort	PTC only	-	78	57/21	45.6	classic 65, follicular variant 12, other 1	2.8	TT+ CLND 139	-	-	8	①②⑤⑦
				PTC+HT	diffuse lymphoplasmacytic infiltrate, oxyphilic cells, formation of lymphoid follicles with germinal centres and atrophic changes in the area of normal thyroid tissue	61	54/7	39.6	classic 43, follicular variant 17, other 1	2.2					
Pilli	2018	Italy	retrospective cohort	PTC only	-	300	209/91	45.1±16.9	-	-	TT 375	-	75.36±46.32	7	④
				PTC+HT	a rich lymphocytic infiltrate diffuse throughout the thyroid gland, commonly organized in follicles with a germinal center.	75	68/7	45.7±14.3							
Qu	2016	China	retrospective cohort	PTMC only	-	886	621/265	44.2±10.6	-	0.77±0.22	TT 84, non-TT 802	T1 782, T3 83, T4 21	63.7±18.6	8	①③④⑤⑥
				PTMC+HT	any one of the following criteria: (1) positive for anti-thyroid peroxidase (TPO) antibody, (2) positive for antithyroglobulin antibody, (3) pathologic confirmation of HT	364	320/44	44.3±10.5		0.72±0.21	TT 39, non-TT 325	T1 337, T3 18, T4 9	61±17.1		
Ryu	2020	Korea	retrospective cohort	PTC only	-	370	293/77	≤55 299, >55 71	-	≤1 230, >1 140	TT + bilateral CLND 850	I 323, II 43, III 4	95.5 (12–158)	8	①③④⑤⑥⑦
				PTC+HT	diffuse lymphocytic infiltration in the area of the normal thyroid tissue irrespective of the presence of anti-thyroid antibodies	480	445/35	≤55 382, >55 98		≤1 352, >1 129		I 444, II 33, III 3			
Singh	1999	USA	retrospective cohort	PTC only	-	331	222/109	median 43	-	median 2	total 158, <total 173	median II	43.6	8	①②③
				PTC+HT	diffuse lymphocytic and plasma cell infiltrate, oxyphilic cells, and the formation of lymphoid follicles and reactive germinal centers	57	45/12	median 41		median 2	total 26, <total 31	median II			
Song	2018	Korea	retrospective cohort	PTC only	-	1064	854/210	median 49.0	-	median 1.2	TT + CLND 1369	-	96	8	①③⑤
				PTC+HT	bilaterally diffuse lymphocytic infiltrates and lymphoid follicles with germinal centres in the area of normal thyroid tissue	305	283/22	median 49.1		median 1.2					
Wang	2018	China	retrospective case-control	PTC only	-	119	91/28	<45 59, ≥45 60	-	1.924±0.993	bilateral thyroidectomies 206	I/II 86, III/IV 33	-	6	①⑥
				PTC+HT	diffuse lymphocytic infiltration in the thyroid parenchyma and stroma, with formation of reactive germinal centers and lymphoid nodules and presence of oxyphilic cells	87	81/6	<45 36, ≥45 51		1.518±1.101		I/II 71, III/IV 16			
Yang	2016	Korea	case-control	PTC only	-	10	-	47(35–59)	conventional 10	0.61(0.1–1.5)	-	-	-	4	①③
				PTC+LT	pathology reports	13			conventional 11, follicular variant 2	0.55(0.2–1.1)					
Ye	2013	China	retrospective case-control	PTC only	-	817	646/171	<30 65, 30–44 362, 45–59 291, >60 99	-	≤1 496, 1–4 304, >4 17	-	I 687, II 25, III 70, IV 35	-	6	①③⑦
				PTC+HT	diffuse lymphocytic and plasma cell infiltration, oxyphilic cells, and lymphoid follicles with reactive germinal centers	187	182/5	<30 23, 30–44 76, 45–59 74, >60 14		≤1 126, 1–4 59, >4 2		I 160, II 4, III 15, IV 8			
Yoon	2012	Korea	case-control	PTC only	-	139	112/27	49.6±11.3	-	0.95±0.60	TT + bilateral CLND 195	-	-	6	①③⑤⑦
				PTC+HT	lymphoid follicles with germinal centers and atrophic changes in the area of normal thyroid parenchyma	56	54/2	45.9±11.1		0.77±0.41					
Zeng	2016	China	retrospective cross-sectional	PTC only	-	397	289/108	45.5±11.8	-	1.57±0.89	thyroidectomy + CLND 619	I/II 240, III/IV 158	-	14	①③⑤⑦
				PTC+HT	diffused lymphoplasmacytic infiltration with germinal centers, parenchymal atrophy with oncocytic changes, and variable amounts of stromal fibrosis throughout the thyroid gland	222	195/27	45.9±12.1		1.43±0.86		I/II 140, III/IV 81			
Zeng	2018	China	case-control	PTC only	-	39	33/6	<45 14, ≥ 45 25	-	-	-	-	-	4	①
				PTC+HT	pathological diagnosis	46	36/10	<45 25, ≥ 45 21							
Zeng	2018	China	cross-sectional	PTC only	-	106	83/23	< 15 14, 15–20 92	-	< 2 25, ≥2 80	thyroidectomy 129	I 98, II 8	-	16	①②③
				PTC+HT	diffuse lymphocytic and plasma cell infiltration in the thyroid parenchyma and stroma, oxyphilic cells, and lymphoid follicles with reactive germinal centers	23	23/0	< 15 3, 15–20 20		< 2 13, ≥2 10		I 23			
Zhang	2014	China	retrospective cohort	PTC only	-	1488	-	46.6±12.4	-	1.34±1.05	unilateral lobectomy with isthmusectomy + cervical LND 1274, unilateral lobectomy of the affected side with isthmusectomy 248, TT + bilateral selective cervical LND 109	I 1228, II–IV 260	36 (8–95)	6	①③④⑤
				PTC+HT	pathological diagnosis	247	220/27	43.1±12.0		1.10±0.77		I 228, II–IV 19			
Zhu	2016	China	retrospective case-control	PTC only	-	486	356/130	≥ 45 237, <45 249	-	≤1 319, >1 167	TT + bilateral CLND 763	-	-	4	①⑤⑦
				PTC+HT	histological diagnosis	277	222/55	≥ 45 125, <45 152		≤1 170, >1 107					
Zhu	2016	China	retrospective cohort	PTC only	-	963	729/234	<45 469, ≥ 45 494	classical 857, other variants 106	1.37±0.92	TT/near TT + 1276 ipsilateral or bilateral CLND	I 625, II 30, III 298, IV 10	105 (3–156)	7	①③⑤⑦
				PTC+HT	pathological diagnosis	313	288/25	<45 155, ≥ 45 158	classical 258, other variants 55	1.32±0.88		I 223, II 12, III 77, IV 1			

**Notes:** QA, Quality assessment; HT, Hashimoto thyroiditis; CLT, chronic lymphocytic thyroiditis; PTC, papillary thyroid cancer; PTMC, papillary thyroid microcarcinoma; FVPC, follicular variant of papillary cancer; ETE, extrathyroidal extension; TT, total thyroidectomy; LND, lymph node dissection; CLND, central-compartment lymph node dissection; LLND, lateral-compartment lymph node dissection; MRND, modified radical neck dissection; TgAb, antithyroglobulin antibodies ① lymph node metastasis ② distant metastasis ③ extrathyroidal extension ④ recurrence ⑤ multifocality ⑥ bilaterality ⑦ invasion ⑧ deaths ⑨ MACIS score ⑩ AMES stage.

### Lymph node metastasis

#### Lymph node metastasis

**Fig 2:**
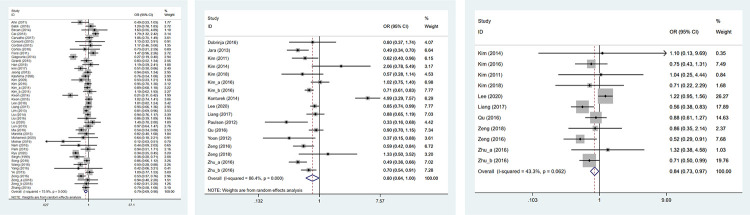
The forest plot of lymph node metastasis between HT group and non-HT group; (a) overall analysis of lymph node metastasis; (b) central lymph node metastasis; (c) lateral lymph node metastasis.

Lymph node metastasis was assessed in 44 studies including 11254 patients. The heterogeneity test results were statistically significant (I^2^ = 75.9%), so the random-effects model was adopted. The result showed that HT group had a lower risk of lymph node metastasis than non-HT group (OR: 0.787, 95%CI: 0.686–0.903, *P* = 0.001) (Table 2, Fig 2A).

**Table 2:** Overall and sensitivity analysis result.

Variables	OR/WMD (95%CI)	*P*	I^2^
**Lymph node metastasis**			
Overall	0.787(0.686,0.903)	0.001	75.9
Sensitivity analysis	0.787(0.686,0.903)		
Publication bias	Z = 0.86	0.39	
**Central lymph node metastasis**			
Overall	0.796(0.636,0.995)	0.045	86.4
Sensitivity analysis	0.796(0.636,0.995)		
Publication bias	Z = 1.52	0.127	
**Lateral lymph node metastasis**			
Overall	0.845(0.733,0.973)	0.02	43.3
Sensitivity analysis	0.845(0.733,0.973)		
Publication bias	Z = 0.78	0.436	
**Distant metastasis**			
overall	0.435(0.279,0.676)	<0.001	0
Sensitivity analysis	0.435(0.279,0.676)		
Publication bias	Z = 0.08	0.938	
**Extrathyroidal extension**			
Overall	0.745(0.657,0.845)	<0.001	74.1
Sensitivity analysis	0.745(0.657,0.845)		
Publication bias	Z = 0.82	0.412	
**Recurrence**			
Overall	0.627(0.483,0.813)	<0.001	16.4
Sensitivity analysis	0.627(0.483,0.813)		
Publication bias	Z = 0.32	0.753	
**Multifocality**			
Overall	1.245(1.132,1.368)	<0.001	61.3
Sensitivity analysis	1.245(1.132,1.368)		
Publication bias	Z = 1.16	0.245	
**Invasion**			
**vascular invasion**			
Overall	0.718(0.572,0.901)	0.004	62
Sensitivity analysis	0.718(0.572,0.901)		
Publication bias	Z = 0.29	0.773	
**Capsular invasion**			
Overall	1.234(0.829,1.835)	0.3	88.5
Sensitivity analysis	1.234(0.829,1.835)		
Publication bias	Z = 0.73	0.466	
**Perineural infiltration**			
Overall	1.922(1.195,3.093)	0.007	0
Sensitivity analysis	1.922(1.195,3.093)		
**Bilaterality**			
Overall	1.394(1.118,1.739)	0.003	78.9
Sensitivity analysis	1.394(1.118,1.739)		
Publication bias	Z = 2.20	0.028	
**Deaths**			
Overall	0.827(0.386,1.773)	0.626	16.8
Sensitivity analysis	0.827(0.386,1.773)		
**Disease-specific death**			
Overall	0.305(0.059,1.585)	0.158	0
Sensitivity analysis	0.305(0.059,1.585)		
**AMES stage**			
**Low risk**			
Overall	1.396(1.109,1.758)	0.005	0
Sensitivity analysis	1.396(1.109,1.758)		
**MACIS score**			
overall	-0.221(-0.306, -0.137)	<0.001	37.8
Sensitivity analysis	-0.221(-0.306, -0.137)		
**<6**			
Overall	1.568(0.930,2.645)	0.092	56.7
Sensitivity analysis		1.568(0.930,2.645)	

**Notes:** OR: odds ratio; WMD: weighed mean difference.

#### Central lymph node metastasis

Seventeen studies involving 7328 patients were identified to assess central lymph node metastasis. The random-effect model result indicated that PTC patients with HT had a lower risk of developing central lymph node metastasis than those without (I^2^ = 86.4%, OR: 0.796, 95%CI: 0.636–0.995, *P* = 0.045) (Table 2, Fig 2B).

#### Lateral lymph node metastasis

A total of 11 studies consisting of 1362 patients provided data to assess lateral lymph node metastasis. The heterogeneity test results were not statistically significant (I^2^ = 43.3%), so the fixed-effect model was adopted. It was shown that HT was associated with a decreasing risk of lateral lymph node metastasis in PTC patients (OR: 0.845, 95%CI: 0.733–0.973, *P* = 0.02) (Table 2, Fig 2C).

### Distant metastasis

**Fig 3:**
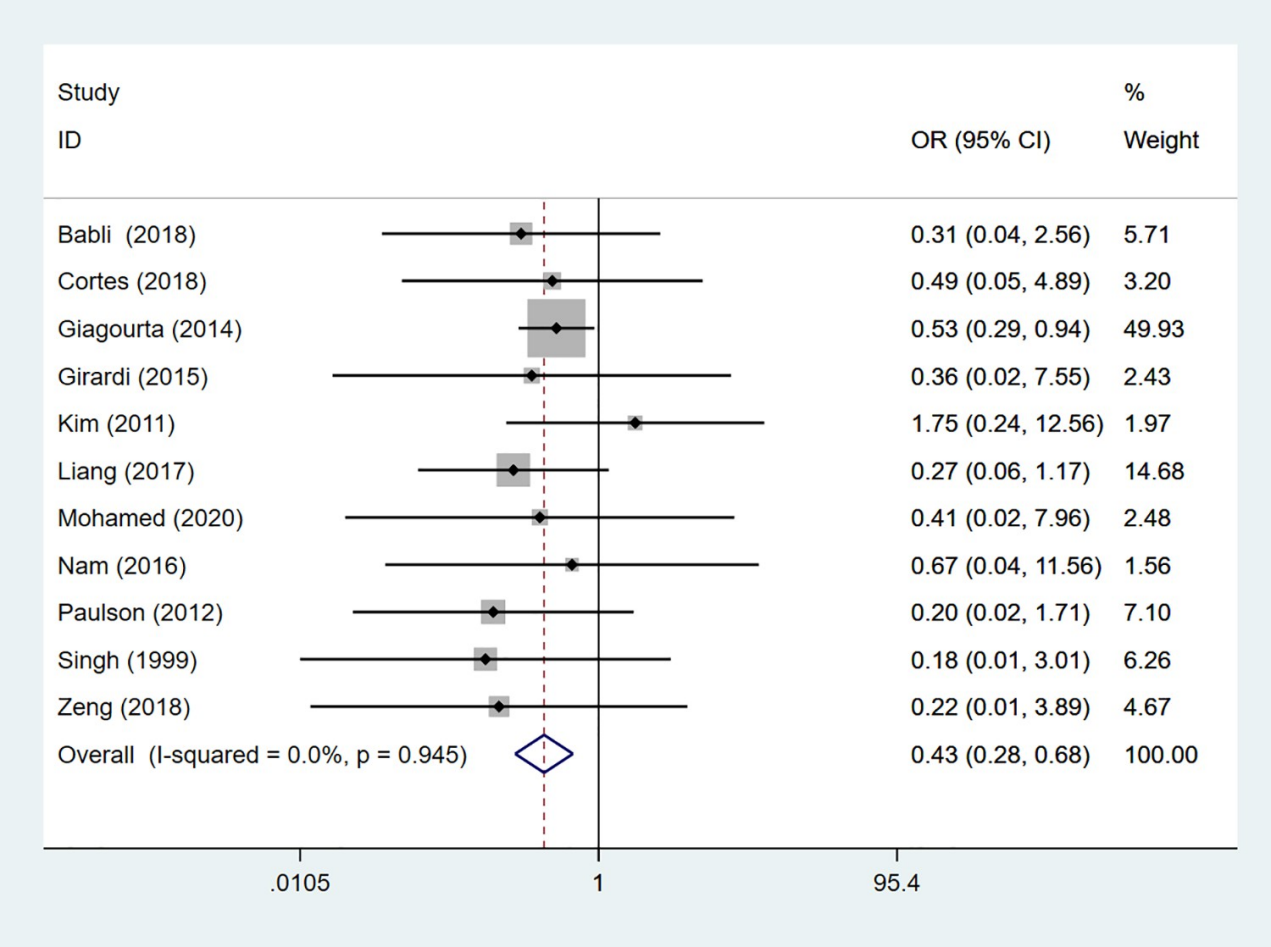
The forest plot of distant metastasis between HT group and non-HT group.

Distant metastasis was assessed in 11 studies comprising 151 patients. The fixed-effects model result showed that the HT group was at a lower risk of distant metastasis than the non-HT group (OR: 0.435, 95%CI: 0.279–0.676, *P*<0.001) (Table 2, Fig 3).

#### Extrathyroidal extension

**Fig 4:**
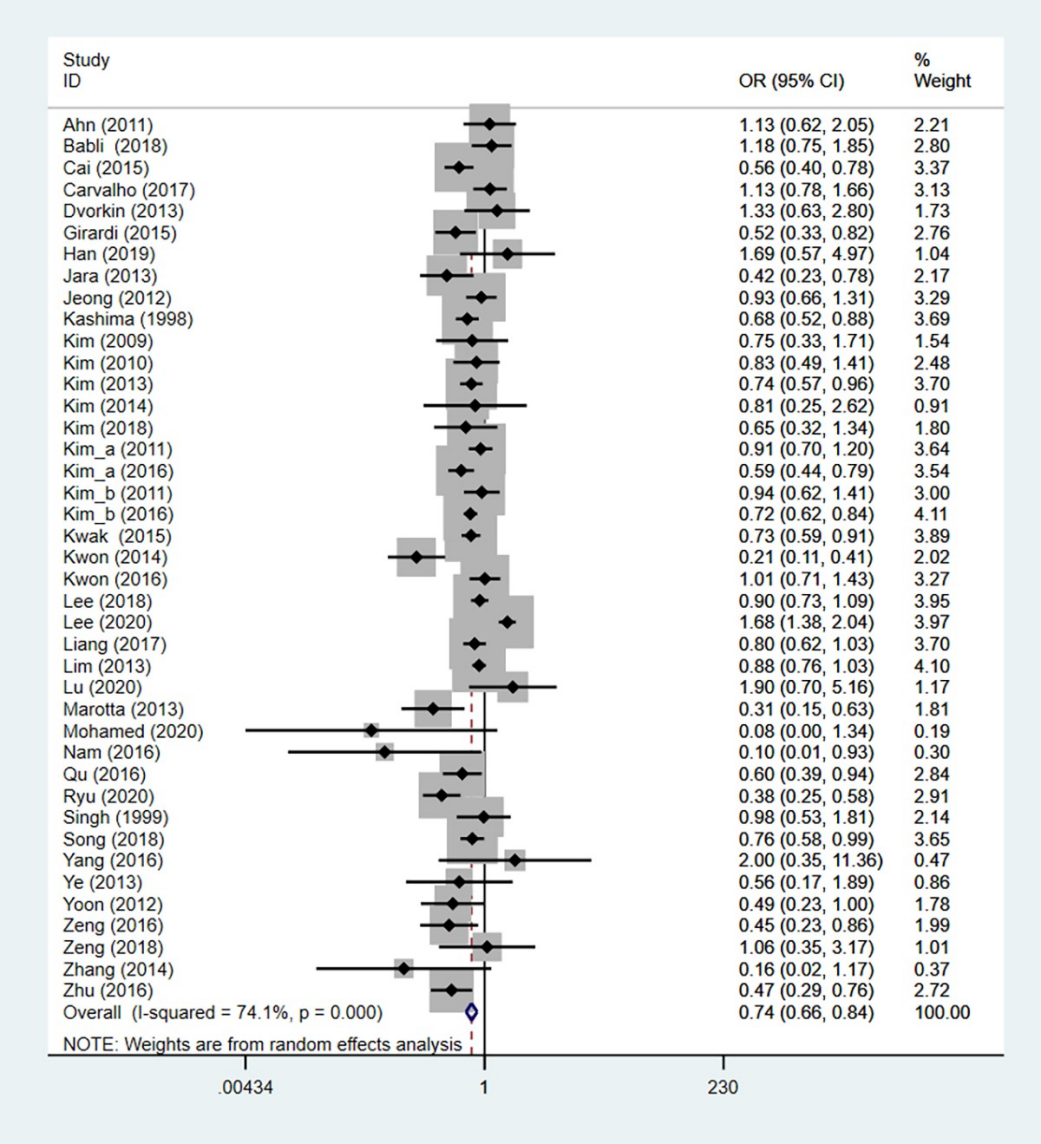
The forest plot of extrathyroidal extension between HT group and non-HT group.

Totally 41 studies covering 13940 patients identified the association between HT and clinical outcome of PTC. The heterogeneity test results were statistically significant (I^2^ = 74.1%), so the random-effect model was utilized. The result revealed that the risk of extrathyroidal extension in the HT group was lower than that in the non-HT group (OR: 0.745, 95%CI: 0.657–0.845, *P*<0.001) (Table 2, Fig 4).

#### Recurrence

**Fig 5:**
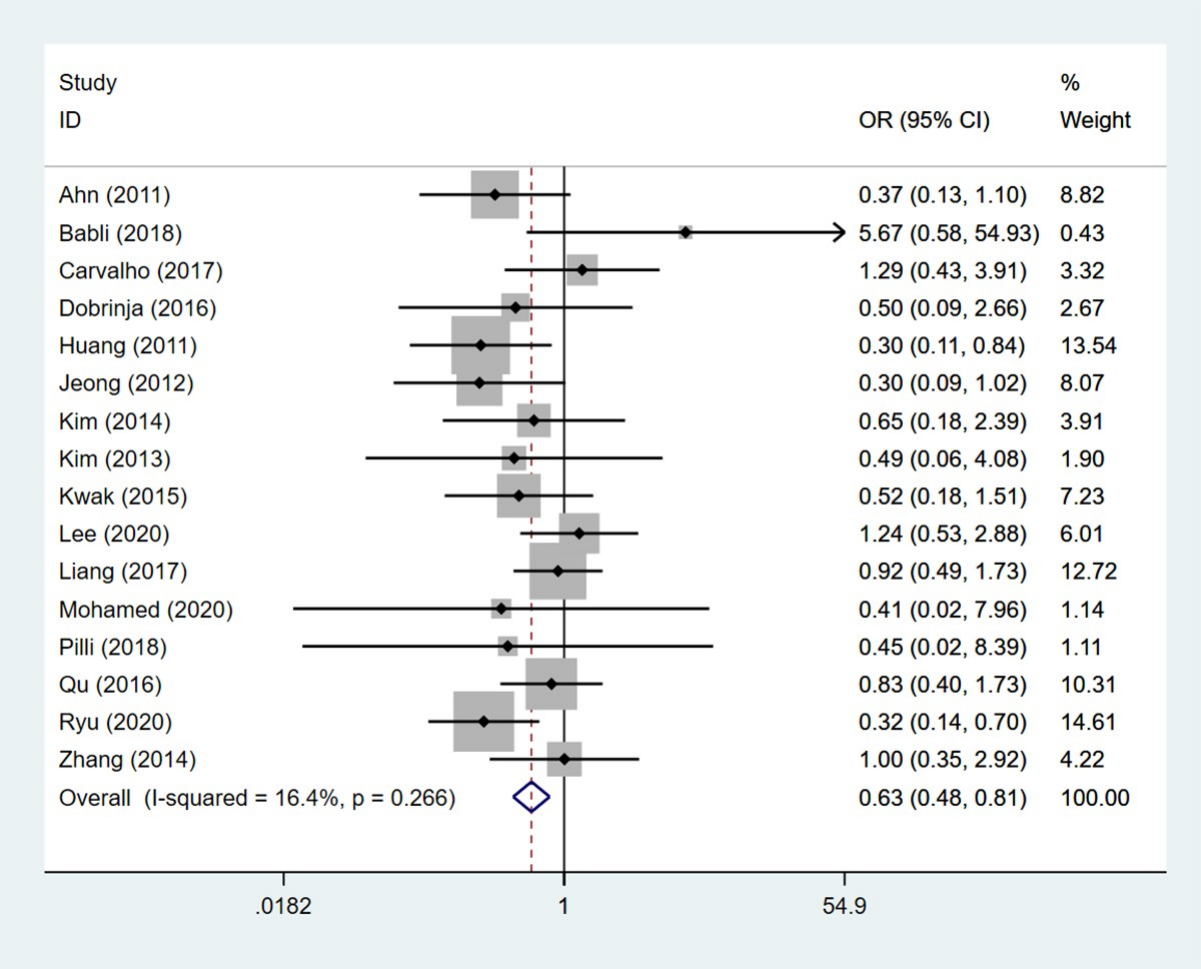
The forest plot of recurrence between HT group and non-HT group.

Sixteen studies containing 577 patients have assessed the recurrence. The result of fixed-effects model demonstrated that HT could decrease the risk of recurrence in PCT (OR: 0.627, 95%CI: 0.483–0.813, *P*<0.001) (Table 2, Fig 5).

#### Multifocality

Multifocality referred to two or more foci found in the same lobe of the gland. A total of 44 studies embracing 10320 were included to evaluate multifocality. The heterogeneity test results were statistically significant (I^2^ = 61.3%), so the random-effects model was used. The result illustrated that that the HT group had a higher risk of multifocality than the non-HT group (OR: 1.245, 95%CI: 1.132–1.368, *P*<0.001) (Table 2).

### Invasion

#### Vascular invasion

**Fig 6:**
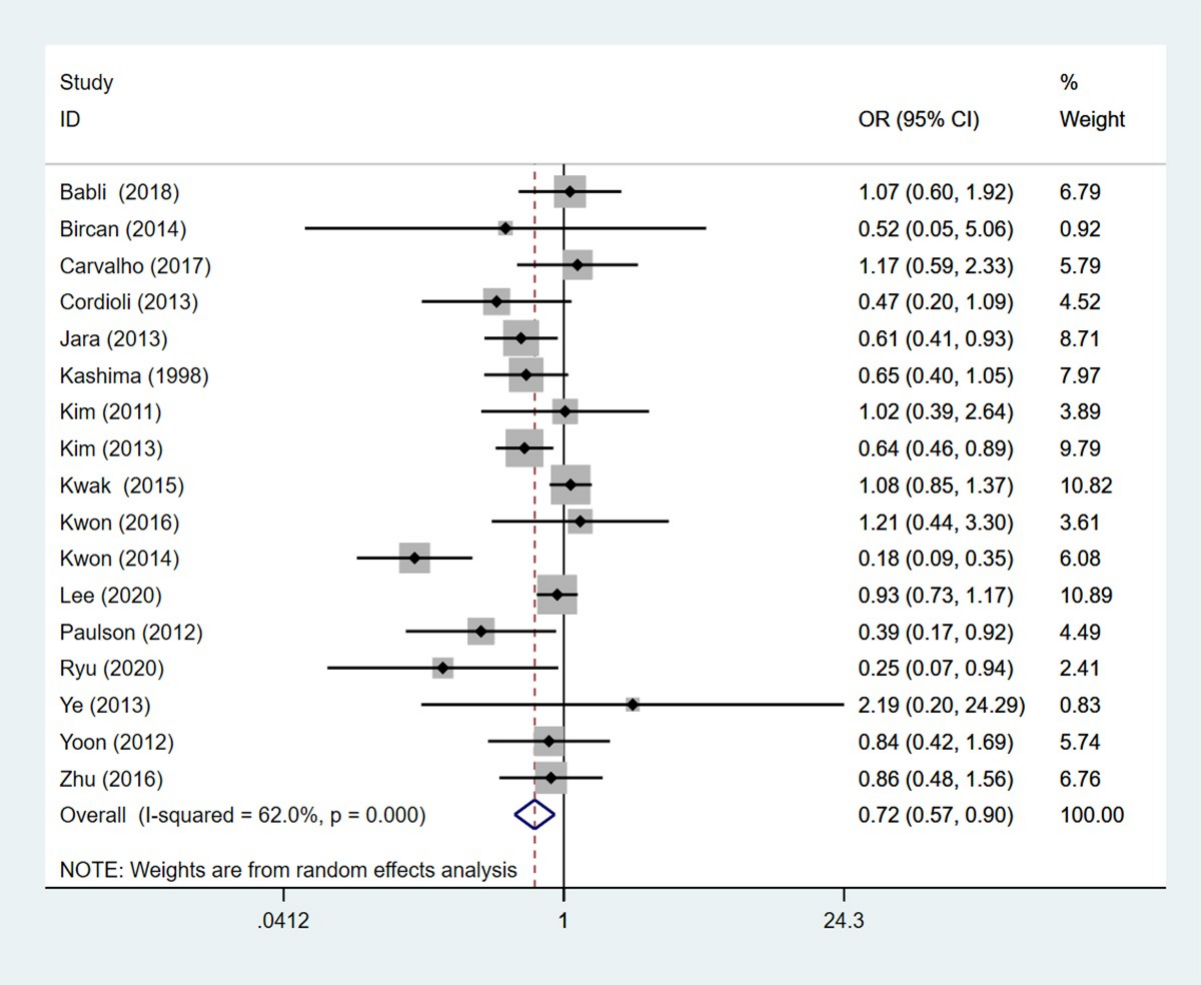
The forest plot of vascular invasion between HT group and non-HT group.

Totally 17 studies embodying 1837 patients probed into the vascular invasion. The result demonstrated that PTC patients with HT had a lower risk of vascular invasion than those without (OR: 0.718, 95%CI: 0.572–0.901, *P* = 0.004) (Table 2, Fig 6).

#### Capsular invasion

Nine studies including 2273 patients assessed the capsular invasion. No difference was found between the HT and non-HT groups in capsular invasion (OR: 1.234, 95%CI: 0.829–1.835, *P* = 0.300).

#### Perineural infiltration

Two studies comprising 132 patients assessed the perineural infiltration.The perineural infiltration risk of the HT group was higher than that of the non-HT group (OR: 1.922, 95%CI: 1.195–3.093, *P* = 0.007) (Table 2).

### Bilaterality

Bilaterality referred to the presence of PTC in both thyroid lobes. Totally 18 studies involving 3421 were enrolled to assess bilaterality. Because the heterogeneity test results were statistically significant (I^2^ = 78.9%), the random-effects model was adopted. The result showed that HT increased the risk of bilaterality in PTC patients (OR: 1.394, 95%CI: 1.118–1.739, *P* = 0.003) (Table 2).

### Deaths

#### Deaths

Death was identified in 6 studies containing 42 patients. There was no statistically significant in death between HT and non-HT groups (OR: 0.827, 95%CI: 0.386–1.773, *P* = 0.626).

#### Disease-specific death

Two studies including 82 patients were included to assess disease-specific death. The result of fixed-effects model demonstrated that HT was not associated with disease-specific death in PTC (OR: 0.305, 95%CI: 0.059–1.585, *P* = 0.158).

### AMES stage-low risk

**Fig 7:**
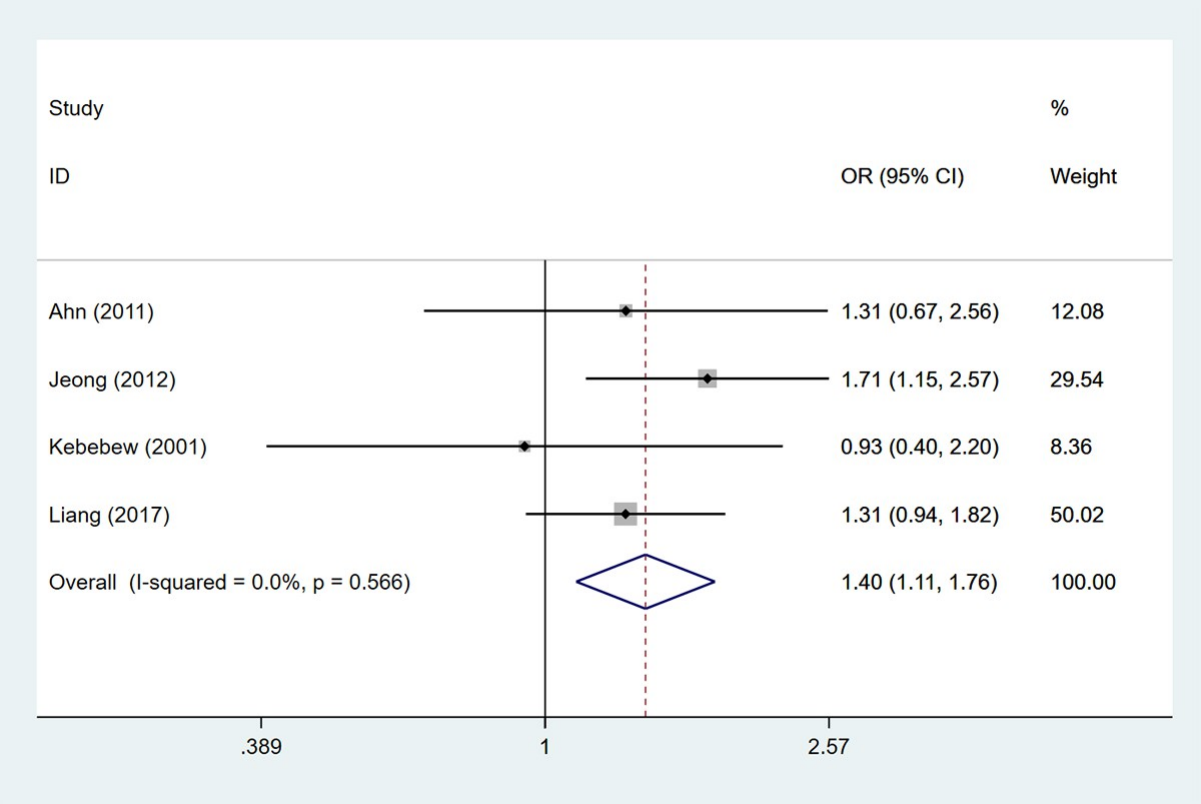
The forest plot of AMES stage-low risk between HT group and non-HT group.

A total of 4 studies embracing 1874 patients were enrolled to assess AMES stage-low risk. The heterogeneity test results showed that the differences were not statistically significant (I^2^ = 0.0%), so the fixed-effects model was used for analysis. The low risk in the AMES stage represents a 20-year survival rate of 99%. The HT group had an advantage over the non-HT group in improving 20-year survival (OR: 1.396, 95%CI: 1.109–1.758, *P* = 0.005) (Table 2, Fig 7).

### MACIS score

#### MACIS score

**Fig 8:**
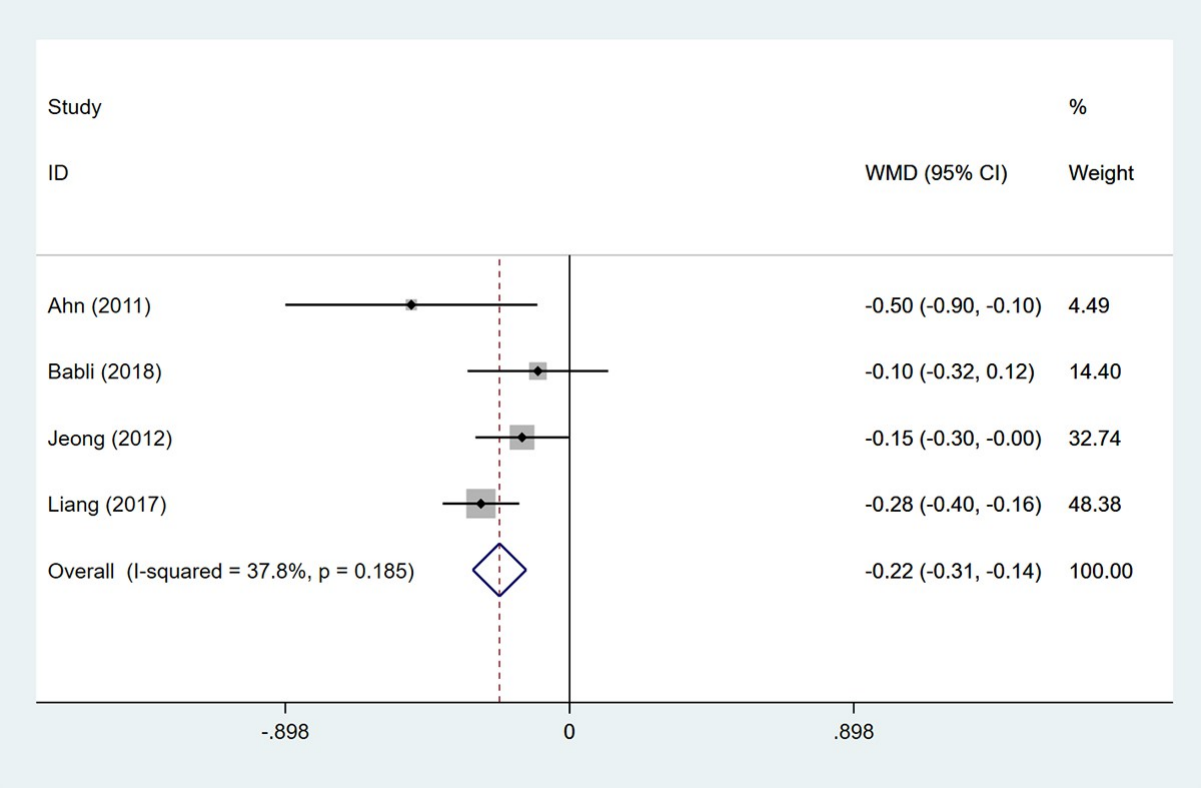
The forest plot of MACIS score between HT group and non-HT group.

The higher the MACIS score, the worse the survival. Four studies involving 2733 patients were included to assess the MACIS score. The result uncovered that the the HT group had an advantage over the non-HT group in improving 20-year survival (WMD: -0.221, 95%CI: -0.306- -0.137, *P*<0.001) (Table 2, Fig 8).

#### MACIS score <6

MACIS score <6 was assessed in 3 studies including 2321 patients. When MACIS score was <6, there was no difference in 20-year survival between HT and non-HT groups (OR: 1.568, 95%CI: 0.930–2.645, *P* = 0.092).

### Publication bias

Begg’s test was used for the assessment of publication bias. The result showed that there was no publication bias for lymph node metastasis (Z = 0.86, *P* = 0.39), central lymph node metastasis (Z = 1.52, *P* = 0.127), lateral lymph node metastasis (Z = 0.78, *P* = 0.436), distant metastasis (Z = 0.08, *P* = 0.938), extrathyroidal extension (Z = 0.82, *P* = 0.412), recurrence (Z = 0.32, *P* = 0.753), multifocality (Z = 1.16, *P* = 0.245), vascular invasion (Z = 0.29, *P* = 0.773), capsular invasion (Z = 0.73, *P* = 0.466) (Table 2). However, there was a publication bias for bilaterality (Z = 2.20, *P* = 0.028) (Table 2). The trim and fill method was applied to adjust data for publication bias. The OR value of the random effects model before the trim and fill method was 1.394 (95%CI: 1.118–1.739). The random effects model was used to estimate the number of missing studies after 7 iterations, and the meta-analysis of all studies was conducted again. The OR value of the random-effects model after the trim and fill method was 2.858 (95%CI: 1.999–3.718), there was no significant change before and after the results, indicating that publication bias had little influence and the conclusions in the literature were relatively robust.

### TSA

#### Lymph node metastasis

A total of 44 articles were included, with a total sample size of 28,813 cases. The required information size (RIS) was 34,021. The estimation of RIS was based on the following variables: Type I error of 0.05, Type II error of 0.2, Power of 80%, Relative Risk Reduction of 20%, and Incidence in Control arm of 10%. The TSA results showed that the cumulative Z curve crossed the traditional boundary line and intersected the TSA boundary line, but did not reach the RIS line, indicating that although the expected sample size was not reached, the positive results were obtained in advance, which further verified that the HT group was better in the low risk of lymph node metastasis than the HT group.

#### Central lymph node metastasis

Seventeen articles with a total sample size of 15947 cases were included, the RIS was 61030 cases, and the RIS was estimated based on the following variables: Type I error of 0.05, Type II error of 0.2, Power of 80%, Relative Risk Reduction of 20%, Incidence in Control arm of 10%. The TSA results showed that the cumulative Z curve crossed the traditional boundary line, but did not reach the TSA boundary line and the RIS line, revealing that the expected sample size was not reached. In the future, more experiments are needed to verify the risk of central lymph node metastasis in the HT group versus the non-HT group.

### Extrathyroidal extension

Forty-one articles were included, with a total sample size of 35,547 cases, and the RIS was 34,408 cases. It was shown that the cumulative Z curve crossed the traditional boundary line, but did not reach the TSA boundary line and the RIS line, indicating that the expected sample size was not reached. More trials are needed in the future to verify the reliability of the conclusion that the HT group has a lower risk of central lymph node metastasis than the non-HT group.

### Recurrence

Sixteen studies were included, with a total sample size of 15,856 cases, and the RIS was 8,342 cases. TSA results demonstrated that the cumulative Z curve crossed the traditional boundary line, intersected the TSA boundary line, and reached the RIS line, indicating that the expected sample size had been reached, and the result was true positive, further verifying that the HT group had a lower risk of recurrence than the non-HT group.

### Multifocality

Concerning multifocality, 44 articles with 34,235 cases were included. The RIS was 20,849 cases. The TSA results illustrated that the cumulative Z-curve crossed the traditional threshold line, intersected with the TSA threshold line, and reached the RIS line, indicating that the expected sample size had been reached. The result was positive, further verifying that the HT group had a higher multifocality risk than the non-HT group.

### Vascular invasion

Seventeen studies were included for vascular invasion, with 14,105 cases sample size, and the RIS was 24,373 cases.The TSA results showed that the cumulative Z-curve crossed the traditional threshold line and intersected with the TSA threshold line, but did not reach the RIS line, indicating that although the expected sample size was not reached, positive results were obtained in advance, further verifying that the risk of vascular invasion in the HT group was lower than that in the non-HT group.

### Bilaterality

Eighteen studies were included to evaluate bilaterality, with a total sample size of 12783 cases, and the RIS was 42465 cases. TSA results showed that the cumulative Z-curve did not reach the TSA threshold line and RIS line, indicating that the expected sample size was not reached. In the future, required to validate the reliability of the conclusion that the risk of bilaterality is higher in the HT group than that in the non-HT group.

## Discussion

No consensus exists on the association between PTC and HT. To resolve this controversy, this study was performed to evaluate the relationship between the two conditions using a meta-analysis. Our analysis revealed that HT was associated with improvements in the clinicopathological characteristics and better prognosis of patients with PTC with lower risk of extrathyroidal extension, lower risk of distant metastasis, lower risk of lymph node metastasis, lower risk of vascular invasion, lower risk of recurrence rate, and a higher 20-year survival rate. Multifocal and bilaterality were positively correlated with HT. Since multifocal and bilaterality are thought to be features associated with PTC development, rather than with its deterioration, these findings are consistent with previous reports of a positive association between HT and PTC development and a protective effect of HT on PTC development [48]. Besides, PTC with HT had a risk of perineural infiltration.

There have been a number of proposed hypotheses to explain the linkage between HT and PTC. From a histological perspective, Tamimi et al. [77] assessed the prevalence and severity of thyroiditis among three types of surgically resected thyroid tumors and found a significantly higher rate of lymphocytic infiltration in patients with PTC. Nevertheless, PTC with concurrent HT is associated with less aggressive disease, less frequent capsular invasion, and less nodal metastasis [22]. Our result supported the result that HT may decrease the risk of lymph node metastasis and vascular invasion in patients with PTC. Similarly, Yoon et al. [70] and Donangelo et al. [78] reported that PTC with HT was significantly associated with a lower incidence of lymph node metastasis.

Furthermore, our findings showed that PTC patients with HT were also less likely to develop recurrence and have a higher 20-year survival rate, which were in agreement with prior studies [41, 66]. Although we did not find the presence of HT indicates lower disease-specific deaths, a recent study by Hu et al. reported that patients with HT had lower rates of tumor recurrence, and lower disease-related mortality compared with patients without HT [79]. Kashima et al. [13] reported a 0.7% cancer specific mortality and a 95% relapse-free 10-year survival rate in patients with HT compared to a 5% mortality and 85% relapse-free 10-year survival rate without chronic thyroiditis. The lymphocytic infiltration of HT may be an immunological response with a cancer-retarding effect, contributing to a favorable outcome of PTC versus other thyroid cancers [80].

Hypotheses about the mechanism of a better prognosis in PTC patients with HT have been evaluated in different ways [17]. HT is a kind of autoimmune disease that leads to the destruction of thyroid follicles through an immune response to a thyroid specific antigen. As PTC cells originating from the follicular cells would express the thyroid specific antigen, auto-antibodies from coexisting HT might destroy the tumor cells in much the same way as in HT alone [81]. Additionally, the infiltrated lymphocytes in patients with PTC are likely to be cytotoxic T cells acting as carcinoma cell killers, secreting interleukin-1 that inhibits thyroid cancer cell growth [82]. In a study on BRAF^V600E^, Xing et al. reported a significantly lower prevalence of BRAF^V600E^ mutation in patients with PTC and HT, suggesting that HT is less likely to be associated with poor prognostic outcomes [83].

Interestingly, we observed that PTC patients with HT were younge than PTC patients without HT. We found that the results among age-balanced were similar to our original outcomes. Nevertheless, in the age-imbalanced groups, there were no differences in lateral lymph node metastasis, extrathyroidal extension, extrathyroidal extension, recurrence, multifocality, and bilaterality between PTC patients with HT and PTC alone. A study by Lun et al. also demonstrated that patients with PTC and HT were younger [56]. Zhang et al. reported older age is a risk factors for BRAF mutation in PTC patients, especially in those without HT [84]. This result suggests that age may be one of the potential sources of bias. More studies are needed in the future with a larger sample size and rigorous design to confirm our findings.

The strengths of the current study need to be mentioned. This was an updated meta-analysis including more studies and more outcomes. There was no apparent publication bias, leading to the research results being more reliable and convincing. Besides, we used TSA to further validate our findings. However, residual confounding variables were a problem. Uncontrolled or unmeasured confounding factors have the potential for bias, and the possibility that residual confounders influenced the results cannot be ruled out. Our analysis was largely limited by the retrospective nature of most of the included studies where clinical details were usually not available. More prospective studies with longer follow-ups are needed to further elucidate this relationship.

## Conclusions

This meta-analysis shows a clinical relationship between two disease entities. PTC patients with HT may have lower incidence of extrathyroidal extension, distant metastasis, lymph node metastasis, vascular invasion, and better prognosis than patients with PTC alone.

## Supporting information

S1 Checklist (DOCX)

S1 File (DOCX)

S2 File (DOCX)
