# Supplementary material for: Association between Hashimoto thyroiditis and clinical outcomes of papillary thyroid carcinoma: A meta-analysis
Source: PLoS One. 2022 Jun 16;17(6):e0269995. doi: 10.1371/journal.pone.0269995 (PMC9202927; doi:10.1371/journal.pone.0269995)
Supplement: S1 File — (DOCX) [file pone.0269995.s002.docx]

The search words from PubMed databases were as follows: “Thyroid Cancer, Papillary” OR “Cancer, Papillary Thyroid” OR “Cancers, Papillary Thyroid” OR “Papillary Thyroid Cancer” OR “Papillary Thyroid Cancers” OR “Thyroid Cancers, Papillary” OR “Thyroid Carcinoma, Papillary” OR “Carcinoma, Papillary Thyroid” OR “Carcinomas, Papillary Thyroid” OR “Papillary Thyroid Carcinomas” OR “Thyroid Carcinomas, Papillary” OR “Papillary Carcinoma Of Thyroid” OR “Papillary Thyroid Carcinoma” OR “Familial Nonmedullary Thyroid Cancer” OR “Nonmedullary Thyroid Carcinoma” OR “Carcinoma, Nonmedullary Thyroid” OR “Carcinomas, Nonmedullary Thyroid” OR “Nonmedullary Thyroid Carcinomas” OR “Thyroid Carcinoma, Nonmedullary” OR “Thyroid Carcinomas, Nonmedullary” AND “Hashimoto Disease” OR “Disease, Hashimoto” OR “Hashimoto Struma” OR “Hashimoto Thyroiditis” OR “Hashimoto Thyroiditides” OR “Thyroiditides, Hashimoto” OR “Thyroiditis, Hashimoto” OR “Hashimoto's Syndrome” OR “Hashimoto Syndrome” OR “Hashimoto's Syndromes” OR “Hashimotos Syndrome” OR “Syndrome, Hashimoto’s” OR “Syndromes, Hashimoto’s” OR “Hashimoto’s Struma” OR “Chronic Lymphocytic Thyroiditis” OR “Chronic Lymphocytic Thyroiditides” OR “Lymphocytic Thyroiditides, Chronic” OR “Lymphocytic Thyroiditis, Chronic” OR “Thyroiditides, Chronic Lymphocytic” OR “Thyroiditis, Chronic Lymphocytic” OR “Hashimoto’s Disease” OR “Disease, Hashimoto’s” OR “Hashimotos Disease” OR “Autoimmune thyroid disease”
