# Supplementary material for: Association between Hashimoto thyroiditis and clinical outcomes of papillary thyroid carcinoma: A meta-analysis
Source: PLoS One. 2022 Jun 16;17(6):e0269995. doi: 10.1371/journal.pone.0269995 (PMC9202927; doi:10.1371/journal.pone.0269995)
Supplement: S2 File — (DOCX) [file pone.0269995.s003.docx]

**Supplementary table of sensitivity analysis of age**

|  | | **Overall** | | | |  | **Age-balanced group (*p*≥0.05)** | | | |  | **Age-imbalanced group** | | | |
| --- | --- | --- | --- | --- | --- | --- | --- | --- | --- | --- | --- | --- | --- | --- | --- |
| Outcomes | Indicators | Number of studies | N (PTC+HT / PTC only) | OR/WMD (95%CI) | *P* |  | Number of studies | N (PTC+HT / PTC only) | OR /WMD (95%CI) | *P* |  | Number of studies | N (PTC+HT / PTC only) | OR (95%CI) | *P* |
| Lymph node metastasis | Overall | 44 | 7774 / 21039 | 0.787 (0.686, 0.903) | 0.001 |  | 27 | 4519 / 10523 | 0.762 (0.644, 0.901) | 0.002 |  | 12 | 2855 / 9471 | 0.757 (0.593, 0.966) | 0.025 |
|  | Central lymph node metastasis | 17 | 4696 / 11251 | 0.796 (0.636, 0.995) | 0.045 |  | 11 | 2011 / 5062 | 0.813 (0.558, 1.184) | 0.28 |  | 6 | 2685 / 6189 | 0.761 (0.600, 0.964) | 0.024 |
|  | Lateral lymph node metastasis | 11 | 2761 / 7003 | 0.845 (0.733, 0.973) | 0.02 |  | 8 | 1364 / 3548 | 0.704 (0.585, 0.847) | <0.001 |  | 3 | 1397 / 3455 | 1.117 (0.894, 1.395) | 0.329 |
| Distant metastasis | | 11 | 1482 / 3468 | 0.435 (0.279, 0.676) | <0.001 |  | 9 | 996 / 2461 | 0.334 (0.162, 0.690) | 0.003 |  | 1 | 441 / 939 | 0.525 (0.295, 0.937) | 0.029 |
| Extrathyroidal extension | | 41 | 10097 / 25450 | 0.745 (0.657, 0.845) | <0.001 |  | 29 | 6498 / 15413 | 0.753 (0.662, 0.856) | <0.001 |  | 10 | 3357 / 9204 | 0.730 (0.518, 1.029) | 0.073 |
| Recurrence |  | 16 | 4263 / 11593 | 0.627 (0.483, 0.813) | <0.001 |  | 12 | 2765 / 8024 | 0.586 (0.433, 0.795) | 0.001 |  | 4 | 1498 / 3569 | 0.763 (0.461, 1.264) | 0.294 |
| Multifocality |  | 44 | 9768 / 24467 | 1.245 (1.132, 1.368) | <0.001 |  | 33 | 6806 / 15884 | 1.306 (1.175, 1.452) | <0.001 |  | 10 | 2733 / 7760 | 1.026 (0.838, 1.256) | 0.803 |
| Bilaterality |  | 18 | 4397 / 8386 | 1.394 (1.118, 1.739) | 0.003 |  | 12 | 1894 / 3834 | 1.502 (1.047, 2.155) | 0.027 |  | 5 | 2436 / 4447 | 1.171 (0.905, 1.515) | 0.229 |
| Invasion | Vascular invasion | 17 | 4393 / 9712 | 0.718 (0.572, 0.901) | 0.004 |  | 12 | 2646 / 6275 | 0.692 (0.490, 0.979) | 0.037 |  | 4 | 1680 / 3332 | 0.807 (0.674, 0.967) | 0.02 |
|  | Capsular invasion | 9 | 1917 / 4804 | 1.234 (0.829, 1.835) | 0.3 |  | 5 | 1304 / 3502 | 1.055 (0.722, 1.541) | 0.783 |  | 3 | 546 / 1197 | 2.575 (2.042, 3.248) | <0.001 |
|  | Perineural infiltration | 2 | 351 / 990 | 1.922 (1.195, 3.093) | 0.007 |  | 2 | 351 / 990 | 1.922 (1.195, 3.093) | 0.007 |  | - | - | - | - |
| Deaths | Deaths | 6 | 1048 / 2933 | 0.819 (0.381, 1.759) | 0.608 |  | 4 | 895 / 2682 | 0.382 (0.110, 1.321) | 0.128 |  | 2 | 153 / 251 | 1.580 (0.028, 87.576) | 0.823 |
|  | Disease-specific death | 2 | 143 / 1914 | 0.305 (0.059, 1.585) | 0.158 |  | 1 | 85 / 1703 | 0.245 (0.034, 1.780) | 0.164 |  | 1 | 58 / 211 | 0.716 (0.034, 15.126) | 0.83 |
| MACIS score | Overall | 4 | 776 / 1957 | -0.221 (-0.306, -0.137) | <0.001 |  | 3 | 718 / 1746 | 0.208 (-0.295, -0.122) | <0.001 |  | 1 | 58 / 211 | -0.500 (-0.898, -0.102) | 0.014 |
| MACIS score | ＜6 | 3 | 419 / 922 | 1.568 (0.930, 2.645) | 0.092 |  | 2 | 361 / 711 | 1.524 (0.671, 3.459) | 0.314 |  | 1 | 58 / 211 | 1.745 (0.867, 3.509) | 0.118 |
| AMES stage | Low risk | 4 | 651 / 1743 | 1.396 (1.109, 1.758) | 0.005 |  | 2 | 552 / 1437 | 1.458 (1.128, 1.884) | 0.004 |  | 2 | 99 / 306 | 1.155 (0.682, 1.956) | 0.592 |

***Notes:** PTC: papillary thyroid carcinoma; HT: Hashimoto thyroiditis; OR: odds ratio; WMD: weighed mean difference; CI: confidence intervals; the effect size of the MACIS score was WMD, the rests were ORs.
